# Long-Term Wavelength Stability of Large Type II FBG Arrays in Different Silica-Based Fibers at High Temperature †

**DOI:** 10.3390/s25061937

**Published:** 2025-03-20

**Authors:** Robert B. Walker, Stephen J. Mihailov, Cyril Hnatovsky, Manny De Silva, Ping Lu, Huimin Ding, Abdullah Rahnama

**Affiliations:** Quantum and Nanotechnologies Research Center, National Research Council of Canada, 100 Sussex Drive, Ottawa, ON K1A 0R6, Canadaping.lu@nrc-cnrc.gc.ca (P.L.); huimin.ding@nrc-cnrc.gc.ca (H.D.); abdullah.rahnama@nrc-cnrc.gc.ca (A.R.)

**Keywords:** high temperature, sensing, fiber Bragg grating, FBG, wavelength drift, thermal stability

## Abstract

Fiber Bragg gratings (FBGs) are useful components in fiber optic sensing systems, which can be highly multiplexed and distributed. In recent years, fabrication using ultrafast lasers has made these devices much more versatile and robust, but questions concerning their high-temperature performance remain. The wavelength resonance of an FBG is naturally sensitive to various parameters of its environment; in particular, changes in the temperature or strain of a fiber tend to induce observable shifts in the Bragg wavelength. Thus, FBGs can offer reliable sensing solutions, provided they are isolated from other influences and their wavelength responses remain well characterized. Nonetheless, it is important to be aware that the isothermal wavelength drift of unstrained FBGs has been previously observed. When this occurs, it can lead to measurement errors and a requirement for sensor recalibration. This study presents a comparison of long-term isothermal wavelength drifts observed at 600 °C, 800 °C, 900 °C and 1000 °C for large numbers of Type II FBGs in different kinds of single-mode fibers. The results provide guidance for the design of high-temperature sensing systems, both in terms of fiber selection and for estimating the maximum time before recalibration becomes necessary to maintain a specified accuracy.

## 1. Introduction

Fiber Bragg gratings (FBGs) are directly affected by changes in strain and temperature through their influences on the grating refractive index and its spatial distribution. This makes FBGs useful for detecting such changes in their environment and for making the monitoring of other phenomena possible with the aid of suitable transducers [1].

The fabrication of FBGs in standard optical fibers gives them a number of desirable characteristics for sensing systems. They are generally small, non-conductive, intrinsically safe, immune to electromagnetic interference (EMI) and easily integrated into large networks and communication systems. Depending on the fibers used, they can also possess significant resistance to mechanical fatigue [2], chemical corrosion [3] and thermal degradation [4].

Since the first report of ultrafast laser-written FBGs in 2003 [5], these devices have become even more versatile. Gratings are now possible and increasingly prevalent in virtually any transparent material [6] and can exhibit a measure of thermal stability approaching their substrate’s glass transition temperature. As such, these devices are well suited for sensing in a variety of extreme environments, including combustion monitoring [7], structural health monitoring of nuclear reactors [8] and temperature monitoring in solar power plants [9].

FBGs are very useful in quasi-distributed sensing systems, primarily because of their ability to be multiplexed, which enables many sensors to be monitored along a single strand of fiber [10]. While more continuously distributed sensing systems are produced using other methods based on Rayleigh, Brillouin and Raman scattering, a drawback of such approaches is that they generally require high levels of data acquisition and averaging. This can produce a trade-off between range and resolution that can affect measurement speed and limit their potential for real-time monitoring. On the other hand, FBGs tend to offer better signal-to-noise ratios and faster interrogation speeds [11].

Type II FBGs in particular are known to have good resistance to erasure at elevated temperatures [4], surviving up to the glass transition temperature of the substrate in which they are written. For this reason, they have been proposed and utilized for a number of high-temperature sensing applications, including external temperature monitoring of an entrained flow gasifier [12], internal temperature monitoring of an oxy-fuel fluidized bed combustor [13], exhaust temperature mapping of a gas turbine combustor simulator [14] and thermal monitoring along the flame tube of a low-emission, high-pressure combustor [15]. All of these installations have performed well over the tested timeframes, with FBGs surviving better than some co-deployed thermocouples. However, they have also been observed to exhibit isothermal wavelength drift over time, even when the induced refractive index change remains relatively stable [16,17]. This has the potential to detrimentally impact sensor accuracy and requires sound understanding to mitigate any adverse effects of this phenomenon, particularly for longer-duration tests at higher temperatures. At lower temperatures, isothermal wavelength drift will be much less severe. In one notable example, 24 regenerated FBGs (RFBGs) were monitored for 758 days at temperatures ranging from 150 °C to 500 °C [18]. Over this time, their drift was at least 13 times less than numbers cited from a 9000 h study at higher temperatures (760 °C to 890 °C) [19].

With very few exceptions [18,19], most of the published thermal stability studies involve tests based on relatively few gratings and short-term monitoring, so the extent of this problem, contributing mechanisms and drift trends at various temperatures are not fully understood, although it is foreseeable that such wavelength drifts can be largely impacted by changes in the underlying waveguide properties themselves [20].

Since the wavelength response of an FBG depends on the effective period of its spatial distribution, changes in the fiber refractive index or length will alter the Bragg resonance. Fibers subjected to high temperatures exhibit refractive index changes through the thermo-optic coefficient, strain-optic coefficient and material modification, including the diffusion of dopants within the fiber. Although fibers may be protected from tensile forces that lead to mechanical strain, thermal strain is produced in response to heating and cooling. The magnitude of thermal strain is a material-dependent property characterized by the coefficient of thermal expansion. For step-index fibers, the core and cladding materials will typically have slightly different thermal expansion coefficients. As a result, changes in temperature will generate a mismatch between the thermal expansion tendencies of the core and cladding. This mismatch generates thermal stresses at the core–cladding interface that can be relieved by glass flow/creep at higher temperatures or frozen in at lower temperatures. The relief of thermal residual stress and other residual stresses induced during the fiber drawing process can alter the fiber length enough to affect an FBG’s wavelength response [4]. Annealing can be used to relieve residual stresses and thereby stabilize sensor performance, but as long as thermal expansion mismatch is present, new thermal stresses will continue to arise in response to temperature change [21].

Because there are many factors at play, in this work, extensive testing was conducted to better assess the potential impact of isothermal drift on the accuracy of our FBG sensing systems over time. Since drift rates depend on temperature, fabrication exposure conditions and the specific waveguide used, data were collected over many months, at a variety of temperatures, for large numbers of gratings written in several different commercially available single-mode fibers, with and without their polyimide and acrylate coatings. Impacts of different writing pulse energies and FBG pre-annealings were also examined. In particular, to assess the minimum achievable isothermal wavelength drift for high-temperature sensing applications, we performed annealing tests on different Type II FBGs within a wide temperature range. Our previous work showed good durability for Type II FBGs near 1000 °C but also indicated that the wavelength drift was an issue [4]. In this work, we studied the wavelength drift at 900 °C and 1000 °C over longer timeframes in a variety of fibers and also examined how the lower annealing temperatures of 600 °C and 800 °C affected the drift.

## 2. Materials and Methods

In this work, we look at 4 different temperatures using large numbers of Type II gratings written in a variety of single-mode fibers, with and without their polyimide and acrylate coatings.

**Figure 1:**
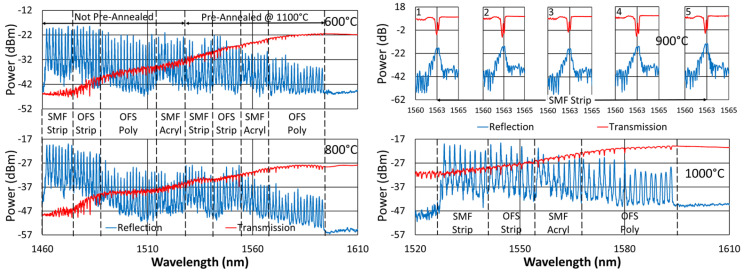
Sample reflection (blue) and transmission (red) spectra of FBGs subjected to long-term annealing at 600 °C, 800 °C, 900 °C and 1000 °C. Gratings were probed from the short-wavelength end and are written in SMF-28 (SMF) and SMT-A1310H (OFS) fibers, through acrylate (Acryl) and polyimide (Poly) coatings, or with the coating stripped (Strip) [23].

A complete description of the 5 gratings tested at 900 °C is provided in Section 5.2 of reference [4]. This group consists of relatively strong FBGs, having the pi-phase shifted, 3rd-order structures shown in the top-right quadrant of Figure 1. Based on the writing conditions and higher broadband loss of these devices, their material modification is expected to consist of nanopores [4,22].

As previous studies had shown good durability for Type II FBGs near 1000 °C, the initial goal of this test was to examine FBG stability at that temperature. After 700 h, wavelength drift was detected, and the temperature was reduced to 900 °C to see if the drift would stabilize. The cited reference includes 380 h of subsequent annealing data at 900 °C, but this test continued for another 2.5 years with the results included here.

**Figure 2:**
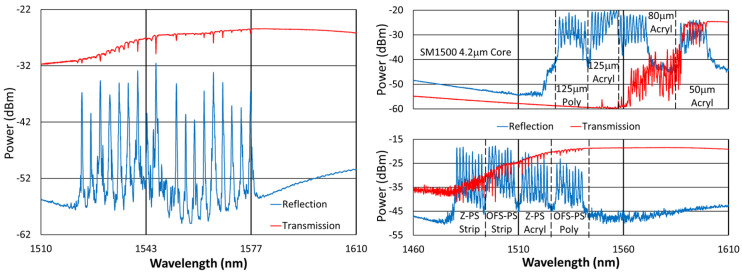
Sample reflection (blue) and transmission (red) spectra of FBGs written in other types of fiber through acrylate (Acryl) and polyimide (Poly) coatings or in stripped fibers (Strip), including OFS A1310 400 µm clad fiber; SM1500 4.2 µm core with 125 µm, 80 µm and 50 µm cladding diameters; and pure silica core SMP-E1550H2 (OFS-PS) and Sumitomo Z Fiber (Z-PS) [31].

Otherwise, our FBGs were fabricated as follows with the sample spectra depicted in Figure 1 and Figure 2. Using our established inscription setup [24], a regeneratively amplified Ti–sapphire laser was used to focus 800 nm, 120 fs pulses through a 12 mm focal-length plano-convex acylindrical lens and a 1st-order phase mask onto the fiber core located ~350 µm after the mask. To accomplish this, a photoluminescence alignment technique based on collecting light perpendicular to the writing beam and fiber axes was used to position the beam focus precisely on the core and avoid damaging the fiber coating [24]. Also, up to 610 µε was applied for tension tuning each inscription [25] to augment the range of Bragg wavelengths writable with the array of phase mask periods used.

The fibers used in this work were selected in order to study the effects of cladding diameter and doping concentration on the thermal drift. FBGs approximately 4 mm in length were written 7 mm apart (center to center) along 9 different single-mode fibers identified in Table 1. Because of their low loss due to few-shot fabrication, their index change is expected to consist of elongated micropores [22].

As specified in Figure 1 and Figure 2, some arrays were written through the fiber coating, acrylate or polyimide, while some were written with the coating removed (room-temperature paint stripper to remove acrylate and 185 °C H_2_SO_4_ to remove polyimide). Typically, these FBGs were produced using 10 pulses or less. To examine the effects of writing pulse intensity, each array had either 9 or 18 FBGs, with each third of an array’s FBGs written using 0.36 mJ, 0.61 mJ or 0.92 mJ pulses, respectively. These writing conditions produced index changes associated with low-loss micropore formation [22].

**Table 1:** Estimated fiber substrate properties.

Fiber	Cladding Diameter	MFD ^1^ @ 1550 nm	Core Ge Doping	Coating
Corning ^2^ SMF-28	125 [26]	10.4 [26]	~4%	Acrylate
OFS ^3^ SMT-A1310H BF04446	125 [27]	10.5 [27]	~4%	Polyimide
Fibercore ^4^ SM1500(4.2/125)P	125 [28]	4.2 [28]	~20% [29]	Polyimide
Fibercore ^4^ SM1500(4.2/125)	125 [28]	4.2 [28]	~20% [29]	Acrylate
Fibercore ^4^ SM1500(4.2/80)	80	4.2 [28]	~20% [29]	Acrylate
Fibercore ^4^ SM1500(4.2/50)	50	4.2 [28]	~20% [29]	Acrylate
Sumitomo Electric ^5^ Z Fiber	125 [30]	10.4 [30]	None	Acrylate
OFS ^3^ SMP-E1550H2	125	10.5	None	Polyimide
OFS ^3^ A1310 400 μm Clad	400	10.5 [27]	~4%	Acrylate

^1^ Mode field diameter; ^2^ Corning, NY, USA; ^3^ Norcross, GA, USA; ^4^ Southampton, UK; ^5^ Osaka, Japan.

**Figure 3:**
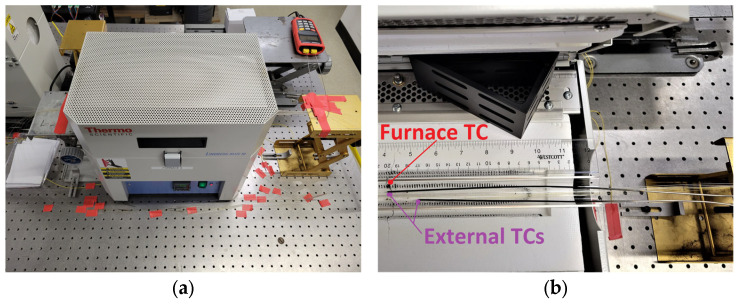
Tube furnace setup showing placement of thermocouples with fibers: (**a**) on the table; (**b**) inside the furnace.

As shown in Figure 3, each group of 9 or 18 FBGs was placed in a Lindberg (Riverside, MI, USA) Blue M Mini Mite TF55035A-1 tube furnace for testing. These gratings were located centrally in the furnace to reduce the effects of any thermal gradients near the periphery. For a 900 °C setpoint, the temperature was measured with an external thermocouple, dropping by 1% over ±3 cm and 2% over ±6 cm with respect to the furnace thermocouple position.

Measurements were made using Omega (Michigan City, IN, USA) Type K thermocouples (KMQXL-062U-24) and Luna (Roanoke, VA, USA) Micron Optics sm125 and si255 interrogators. To use the equipment efficiently, composite arrays of multiple fibers were spliced together in series with FBGs arranged from short wavelength to long wavelength and reflection measurements made from the short-wavelength end of each fiber.

Generally, FBGs pre-annealed at higher temperatures are more stable at lower temperatures. For the remaining studies, 1100 °C (maximum operating temperature of these tube furnaces) was selected as the pre-annealing temperature to examine whether or not it would have a beneficial impact on wavelength stability, particularly at 1000 °C. The duration of pre-annealing was chosen in order to prevent excessive erasure of the FBGs at that temperature. The FBGs that were subjected to pre-annealing were first heated to 1100 °C for ~10 h (600 °C and 800 °C tests) or ~18 h (1000 °C test). They were next cooled to room temperature before heating back up to their designated operating temperatures.

## 3. Results

The results obtained for testing at each temperature are presented in Section 3.1, Section 3.2, Section 3.3 and Section 3.4 below. For each temperature, spectral data are shown first, captured at various times throughout each test. Data trends over time, collected by the thermocouples and interrogator, are given next. Finally, more processed data are used to examine relevant details and trends in the measured responses at each temperature.

### 3.1. 1000 °C Test

#### 3.1.1. Spectral Data

**Figure 4:**
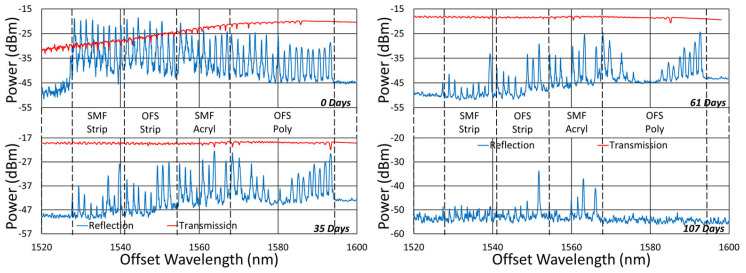
Reflection (blue) and transmission (red) spectra of FBGs subjected to 1000 °C annealing written in SMF-28 (SMF) and SMT-A1310H (OFS) fibers through acrylate (Acryl) and polyimide (Poly) coatings or with the coating stripped (Strip).

Beginning with the gratings subjected to prolonged annealing at 1000 °C, Figure 4 presents spectral data obtained from the SMF-28 and SMT-A1310H fibers at various points during the test. At this temperature, significant, progressive erasure of these FBGs is observed over time.

**Figure 5:**
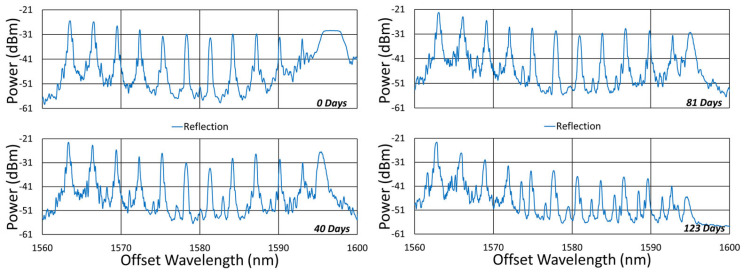
Reflection plots of FBGs subjected to 1000 °C annealing written in stripped OFS A1310 400 µm clad fiber.

Similarly, the spectral data obtained for the 1000 °C annealing of the FBGs in the OFS A1310 400 µm clad fiber are shown in Figure 5. In this case, the erasure is not nearly as strong, although it is still present.

The initial spectra were measured at room temperature, while the others were measured at 1000 °C. For easy comparison, their wavelength axes were offset to compensate for the temperature-induced wavelength shift.

#### 3.1.2. Temperature and Peak Data

**Figure 6:**
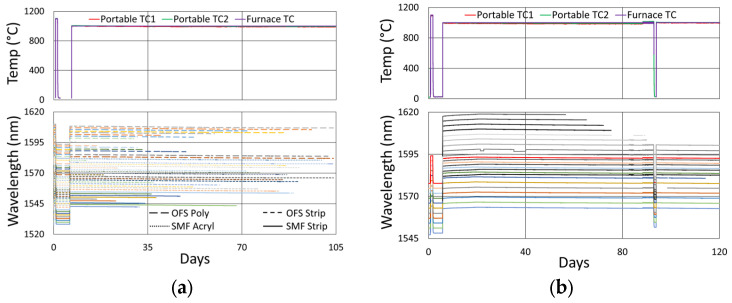
Temperature and peak wavelength data over time for 1000 °C annealing tests in (**a**) SMF-28 (SMF) and SMT-A1310H (OFS) fibers written through acrylate (Acryl) and polyimide (Poly) coatings or with the coating stripped (Strip) and (**b**) stripped OFS A1310 400 µm clad fibers.

**Figure 7:**
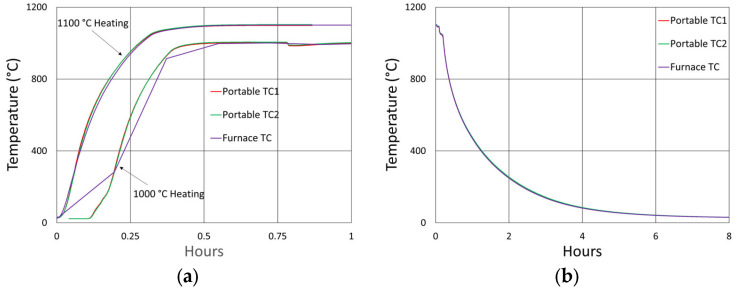
The 1000 °C test (**a**) transient heating rates for 1100 °C pre-annealing and 1000 °C extended testing and (**b**) transient cooling rate following 1100 °C pre-annealing.

In Figure 6, the temperature and peak wavelength data trends are shown, with the 1100 °C pre-annealing phase clearly distinguishable from the extended testing phase at 1000 °C. The wavelength peak data, which were extracted automatically from the spectral data by the FBG interrogator, also clearly illustrate some progressive erasure of FBGs as various peaks lose visibility and their lines disappear from the plot. Figure 7 plots transient heating and cooling data associated with the pre-annealing and extended testing phases.

#### 3.1.3. Details and Trends

**Figure 8:**
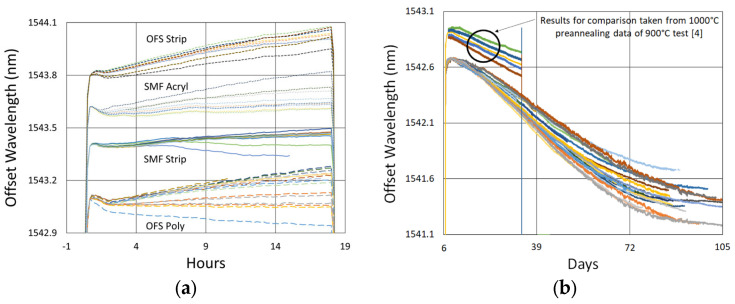
Drift trends of SMF-28 (SMF) and SMT-A1310H (OFS) fibers written through acrylate (Acryl) and polyimide (Poly) coatings or with the coating stripped (Strip) during (**a**) 1100 °C pre-annealing and (**b**) 1000 °C extended testing.

**Figure 9:**
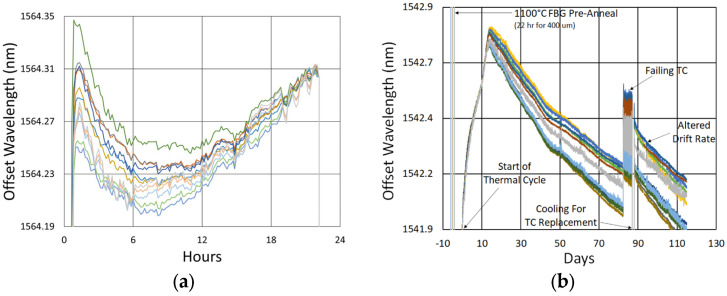
Drift trends of OFS A1310 400 µm clad fibers during (**a**) 1100 °C pre-annealing and (**b**) 1000 °C extended testing.

In Figure 8 and Figure 9, the wavelengths of each device are offset for comparison. Variability is observed between the wavelength drift of different devices, both during pre-annealing and also during extended testing. These differences were not clearly linked to writing pulse energy, coating condition, etc., but likely result from nonuniformities in device fabrication or furnace temperature, as previously demonstrated by Laffont et al. [8].

More generally, one can see that the initial wavelength drift tends toward higher values before turning to a more gradual and prolonged blue drift, something that has been noted in the literature before [4].

In Figure 8b, one can also see how the gratings fabricated in [4] (the basis of our 900 °C test results) compare with those fabricated here. While at 1000 °C, the drift looks similar over the 700 h tested, the average drift slope is slightly less for the gratings fabricated in [4]. This a consequence of greater nanopore-related disruption, with the gratings in [4] written using 15–20 pulses, in contrast with the few pulse gratings fabricated here.

In Figure 9a, one can see that during pre-annealing, the wavelength drift of the 400 µm clad fiber is substantially more non-linear than that shown in Figure 8a for the other fibers. In Figure 9b, a significant discontinuity and ripple is observed around the 85 day mark. This is caused by an oscillating furnace temperature, resulting from a failing furnace thermocouple. The furnace was cooled down to room temperature in order to replace this thermocouple. It is interesting to note that when the test resumed, the drift rate appears to have changed.

**Figure 10:**
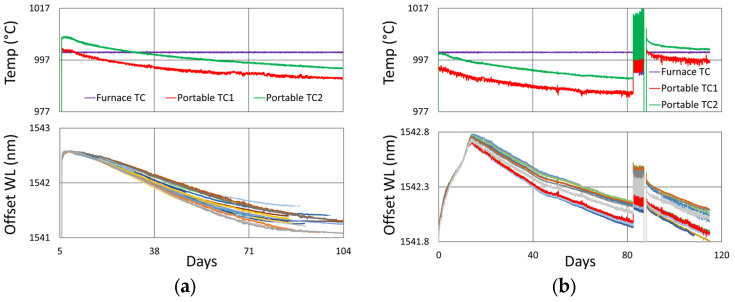
Relative thermocouple drift, for 1000 °C annealing tests of (**a**) SMF-28 and SMT-A1310H fibers and (**b**) OFS A1310 400 µm clad fibers.

In Figure 10, the thermocouple drift is plotted for comparison. The furnace setpoint is maintained by its controller relative to the furnace thermocouple, so it appears to be drift-free, although its error is expected to be similar to that of the external thermocouples.

The measured drift of the external thermocouples, in both cases, appears to be consistent, indicating a drift to lower temperatures over time relative to the furnace thermocouple. Although the long-term trend exhibits a similar pattern to the wavelength drift of the FBGs in SMF-28 and SMT-A1310H, it is most definitely different for the OFS A1310 400 µm clad fibers. In the standard fiber, the temperature-induced wavelength shift has been previously calibrated and shown to be ~14.4 pm/°C at 1000 °C [12]. At this temperature, the reported thermocouple drift (~12 °C) corresponds to a wavelength shift of 173 pm, which is much less than the isothermal wavelength drifts observed (~1 to 1.5 nm). This confirms that these wavelength drifts are not explained by temperature change.

### 3.2. 900 °C Test

#### 3.2.1. Spectral Data

**Figure 11:**
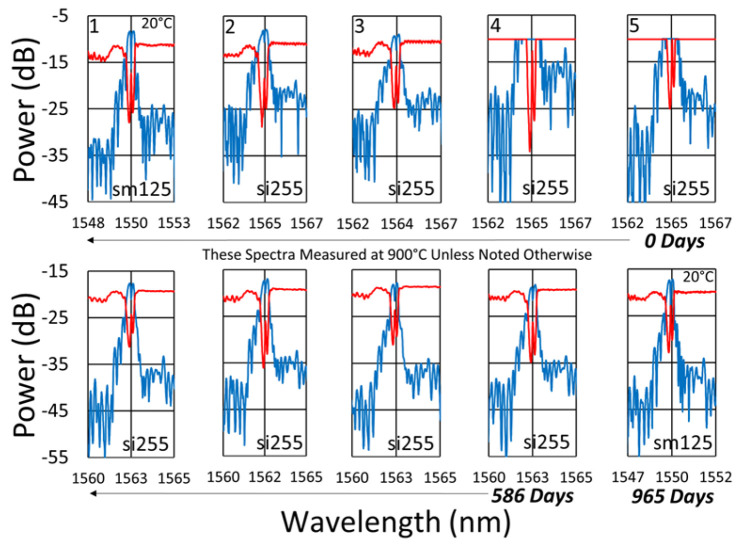
Reflection (blue) and transmission (red) spectra of FBGs subjected to 900 °C annealing written in stripped SMF-28 fibers recorded using Micron Optics sm125 and si255 interrogators.

For performance at 900 °C, we look to results extending from the study mentioned in Section 2 [4]. Recall that for this set of gratings, the fabrication conditions were slightly different. The gratings were much stronger, all made with the same Bragg wavelength, using a third-order mask, and they contain pi-phase shifts. Examining the spectral data at different times during the test (Figure 11), on average, one observes that at this temperature only slight erasure has occurred, even after 19 to 32 months of annealing.

#### 3.2.2. Temperature and Peak Data

**Figure 12:**
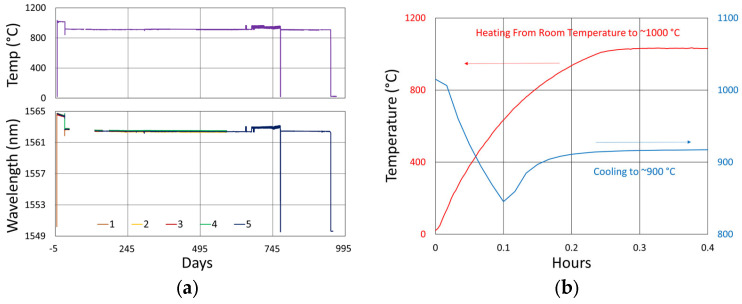
The 900 °C annealing test: (**a**) temperature and peak wavelength data over time; (**b**) transient heating for initial ramp to 1000 °C (red) and transient cooling to 900 °C (blue).

Figure 12a plots the temperature and peak wavelength data trends with the initial 1000 °C pre-annealing phase followed by extended testing at 900 °C. Both the thermocouple and wavelength peak data indicate similar trends. Nonetheless, close examination of the plot slopes reveals that early in the test, during 1000 °C pre-annealing, the wavelength drifts noticeably change, although the temperature remains stable. Figure 12b plots the transient heating up to 1000 °C for pre-annealing and cooling to 900 °C for extended testing.

#### 3.2.3. Details and Trends

**Figure 13:**
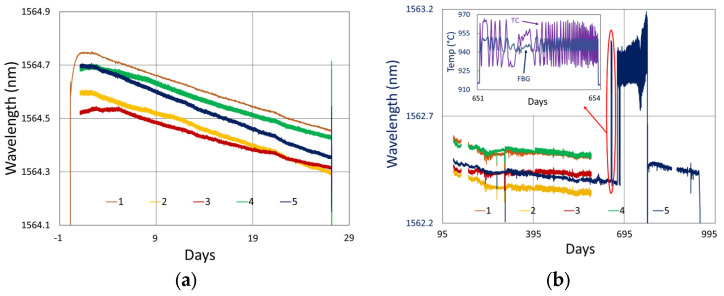
Drift trends during (**a**) 1000 °C pre-annealing and (**b**) 900 °C extended testing.

Looking closely at the pre-annealing data in Figure 13a, once again, an initial tendency towards red drift is observed, followed by a more gradual blue drift, which persists over the remainder of the test.

During the latter part of the test, shown in Figure 13b, only one of the devices was monitored in real time since the other interrogator channels were needed for other experiments. Nevertheless, measurements made at the end of the test confirmed that all of the FBGs survived the experiment with comparable reflectivity and wavelength drift.

In Figure 12 and Figure 13b, a significant temperature fluctuation is observed. This resulted from a failing furnace thermocouple, as is also observed in Figure 9b and Figure 10b. The furnace’s fluctuating response to inaccurate temperature feedback was allowed to continue in order to see how well it could be tracked by the FBGs. Eventually, the thermocouple was replaced at room temperature before resuming stable operation at 900 °C for several more months. Once again, as noted in Section 3.1.3, the rate of blue drift appears to have increased slightly following thermocouple replacement.

**Figure 14:**
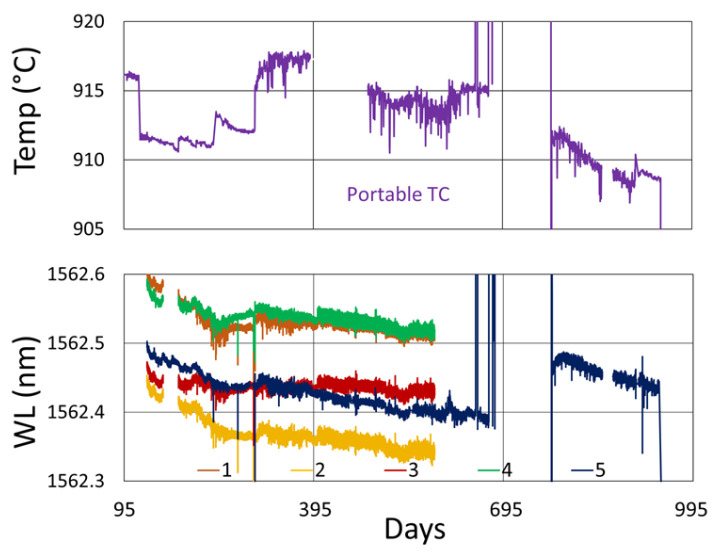
Relative thermocouple drift during extended testing for 900 °C annealing test.

In Figure 14, the thermocouple drift for this test is plotted. In this case, no data were captured from the furnace control thermocouple, but data were recorded using one external thermocouple. During extended testing, the thermocouple-reported temperature variation was limited to ~8 °C. This does not match the wavelength drift trends at all times, but for this test it is of sufficient magnitude to produce much of the observed wavelength drift (~0.1 nm).

### 3.3. The 800 °C Test

#### 3.3.1. Spectral Data

**Figure 15:**
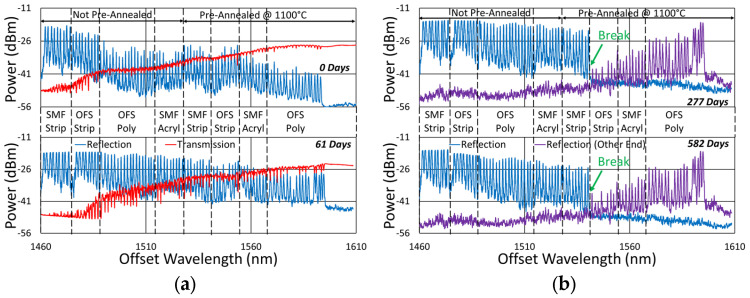
Reflection (blue and purple) and transmission (red) spectra of FBGs subjected to 800 °C annealing written in SMF-28 (SMF) and SMT-A1310H (OFS) fibers through acrylate (Acryl) and polyimide (Poly) coatings or with the coating stripped (Strip): (**a**) unbroken fiber; (**b**) broken fiber.

At 800 °C, a break in the fiber occurred during the experiment. Although we were able to continue monitoring the severed gratings from the long-wavelength end, as shown in Figure 15b, their cumulative cladding mode loss became increasingly problematic as more and more gratings were probed.

**Figure 16:**
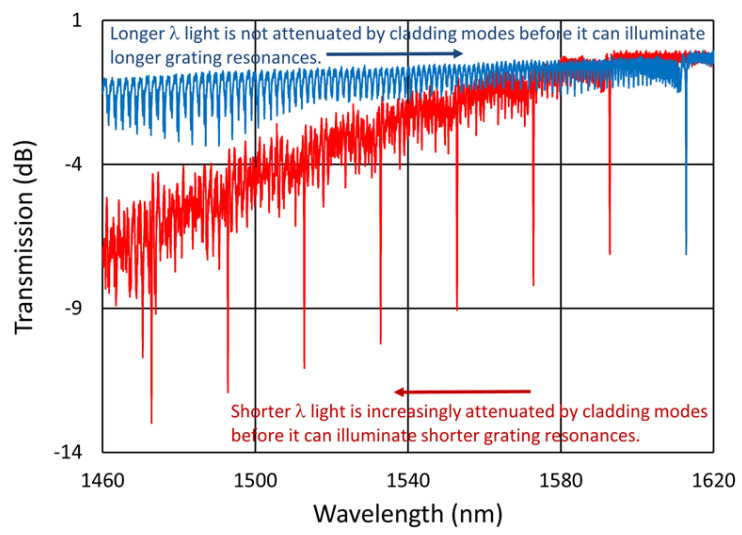
Illustration of FBG transmission loss accumulation based on an individual representative FBG spectrum (blue) offset by 20 nm and superimposed incoherently with itself to estimate the transmission loss of 7 FBGs in series (red). Interrogating ascending wavelength resonances in reflection from the short-wavelength fiber end ensures that the cladding mode resonances do not obscure the Bragg resonances, whereas peaks monitored in transmission or illuminated from the long-wavelength fiber end will be obscured by the accretion of cladding mode losses at the same wavelength.

We have known about this phenomenon for some time and have highlighted its importance recently [7]. The benefit of arranging grating resonances within an array from short wavelength to long wavelength and probing from the short-wavelength end of the fiber is shown in Figure 16. When probing from the short-wavelength end, accumulated cladding mode loss does not affect the longer-wavelength light needed to probe Bragg resonances further along the fiber, whereas when probing from the long-wavelength end, the light needed to illuminate shorter-wavelength resonances is already attenuated by the cladding modes of longer-wavelength gratings, obscuring their illumination and any reflected signal.

#### 3.3.2. Temperature and Peak Data

**Figure 17:**
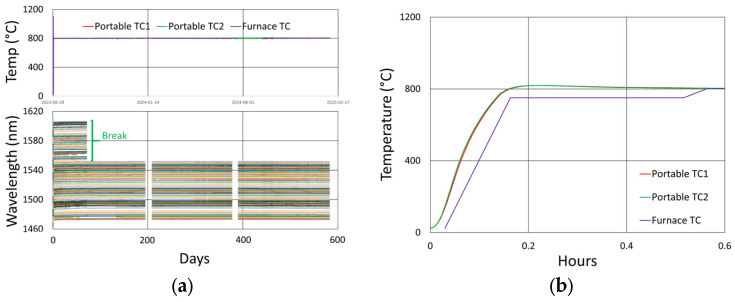
The 800 °C annealing test: (**a**) temperature and peak wavelength data over time; (**b**) transient heating for 800 °C extended testing.

Temperature and peak wavelength trends for this test are shown in Figure 17a, including 1100 °C pre-annealing for some devices, followed by extended testing at 800 °C. Consequences of the fiber break are also apparent in the peak data, as 40% of the Bragg resonances were no longer trackable from the short-wavelength end of the fiber. Heating and cooling rates for the pre-annealing may be obtained from Figure 7, while Figure 17b plots the transient heating data associated with the extended testing phase.

#### 3.3.3. Details and Trends

**Figure 18:**
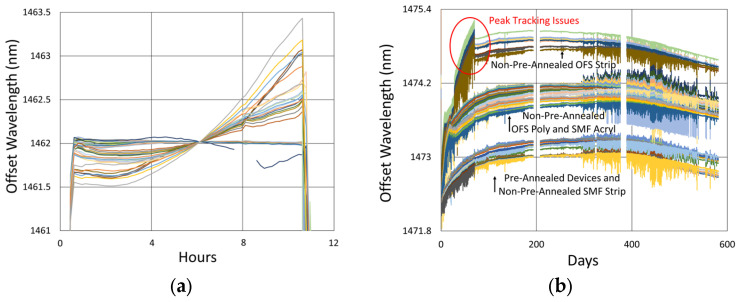
Drift trends of FBGs written in SMF-28 (SMF) and SMT-A1310H (OFS) fibers through acrylate (Acryl) and polyimide (Poly) coatings or with the coating stripped (Strip): (**a**) 1100 °C pre-annealing; (**b**) 800 °C extended testing.

In Figure 18, variability is again observed between the wavelength drift of different devices, both during pre-annealing and also during extended testing. Once again, these differences had no clear link to writing pulse energy, number of pulses, coating condition, etc., but are attributed to nonuniformities in device fabrication or temperature, as discussed in Section 3.1.3. Slight differences are observable for the various fiber configurations early on, with the circled discontinuity in Figure 18b likely due to some sort of interrogator peak tracking issue.

Unlike the results obtained at higher temperatures, it is apparent that in this case, the red-drift to blue-drift transition does not occur until 200 to 400 days after the start of extended testing.

**Figure 19:**
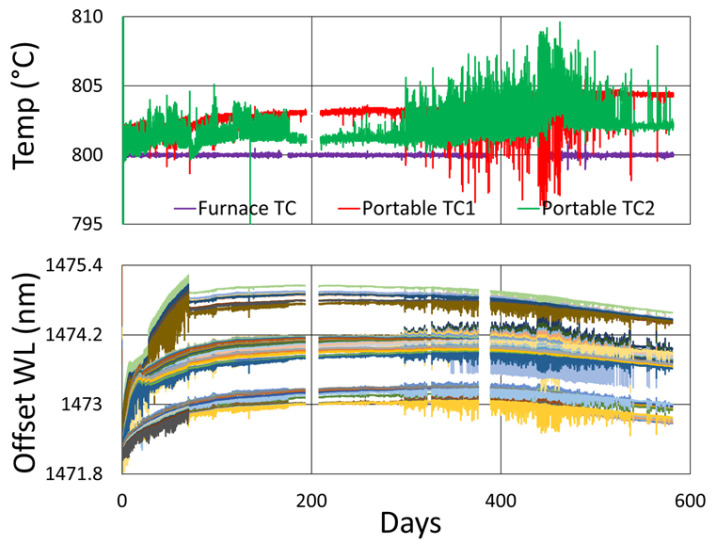
Relative thermocouple drift for 800 °C annealing test.

Figure 19 plots the thermocouple drift for this test. Once again, the furnace setpoint is maintained by its controller relative to the furnace thermocouple, so as in Figure 10, it appears to be drift-free, although its error should be similar to the external thermocouples.

In this case, the measured drift of both external thermocouples trends to higher temperatures over time with respect to the furnace thermocouple. This also reflects the wavelength drift trend of the FBGs, but as discussed in Section 3.1.3, the average thermocouple drift (~5 °C) remains much less than that needed to produce the observed isothermal wavelength drift (~1 to 1.5 nm), again confirming that temperature change does not explain this wavelength drift. A significant thermal jitter is also present. Since this appears in the FBG wavelength data as well, it likely results from real fluctuations of the furnace temperature, possibly due to degradation of the furnace thermocouple or some other issue affecting the furnace temperature controller.

### 3.4. 600 °C Test

#### 3.4.1. Spectral Data

**Figure 20:**
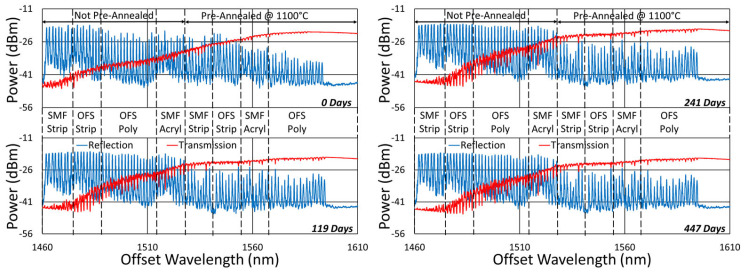
Reflection (blue) and transmission (red) spectra of FBGs subjected to 600 °C annealing written in SMF-28 (SMF) and SMT-A1310H (OFS) fibers through acrylate (Acryl) and polyimide (Poly) coatings or with the coating stripped (Strip).

**Figure 21:**
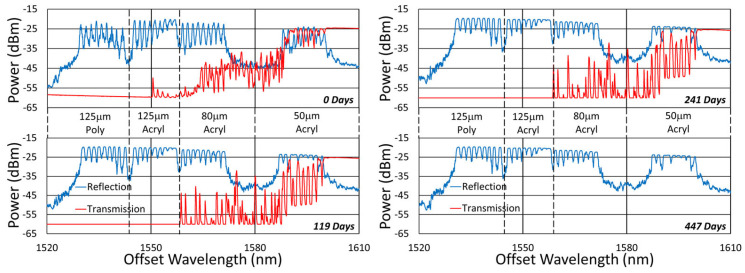
Reflection (blue) and transmission (red) spectra of FBGs subjected to 600 °C annealing written in SM1500 4.2 µm core fibers with 125, 80 and 50 μm cladding diameters through acrylate (Acryl) and polyimide (Poly) coatings.

**Figure 22:**
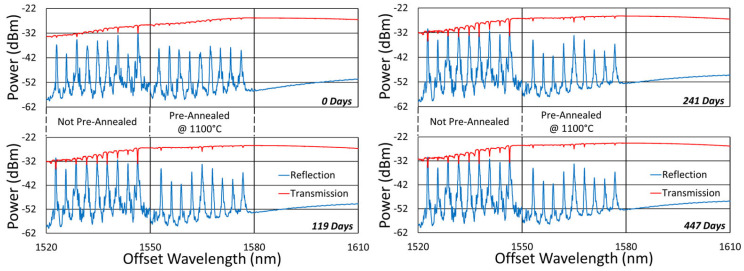
Reflection (blue) and transmission (red) spectra of FBGs subjected to 600 °C annealing written in stripped OFS A1310 400 µm clad fiber.

**Figure 23:**
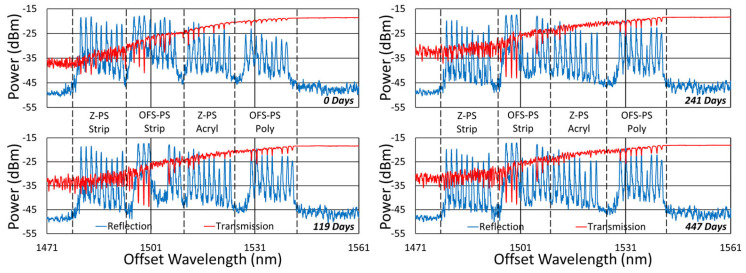
Reflection (blue) and transmission (red) spectra of FBGs subjected to 600 °C annealing written in pure silica core SMP-E1550H2 (OFS-PS) and Sumitomo Z Fiber (Z-PS) through acrylate (Acryl) and polyimide (Poly) coatings or with the coating stripped (Strip).

Figure 20, Figure 21, Figure 22 and Figure 23 present spectral data from various points during the 600 °C tests. In this case, although some slight changes are apparent in the spectra, for all of the fibers tested, grating reflectivity remained strong and relatively consistent throughout, even after a year of testing. Such changes have primarily occurred early on and include both subtle growth and the erasure of resonance peaks.

In Figure 21, a failure of the 50 µm fiber, at some time between 241 and 447 days, obscured the transmission measurement, although monitoring in reflection remains unaffected.

#### 3.4.2. Temperature and Peak Data

**Figure 24:**
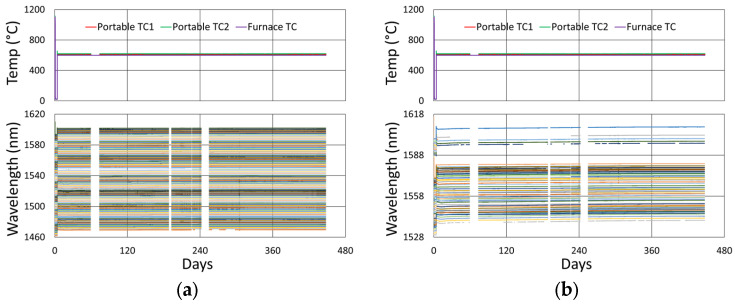
Temperature and peak wavelength data for 600 °C tests of FBGs in (**a**) SMF28 and SMT-A1310H fibers and (**b**) SM1500 4.2 µm core fibers.

**Figure 25:**
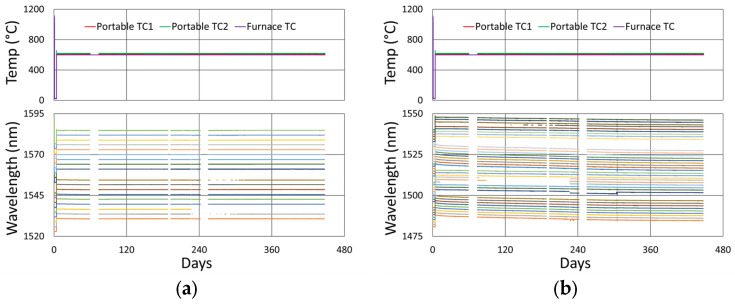
Temperature and peak wavelength data for 600 °C tests of FBGs in (**a**) stripped OFS A1310 400 µm clad fiber and (**b**) pure silica core SMP-E1550H2 and Sumitomo Z Fiber.

**Figure 26:**
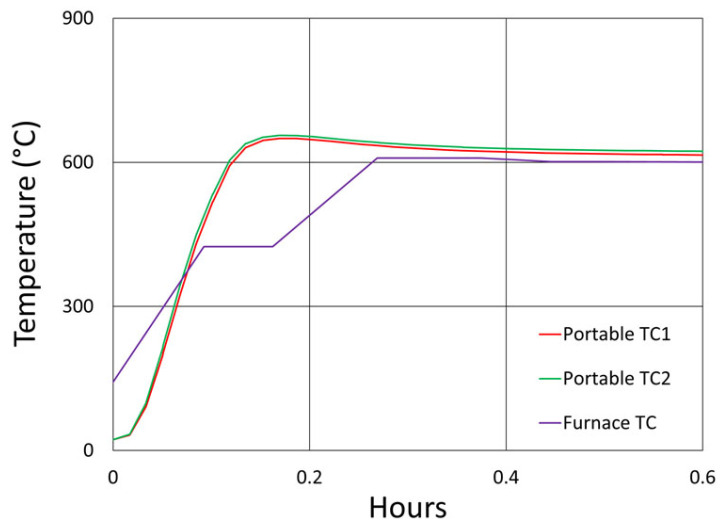
Transient heating rates for extended testing at 600 °C.

Figure 24 and Figure 25 provide temperature and peak wavelength trends for these tests, including 1100 °C pre-annealing for some devices followed by extended testing at 600 °C. The heating and cooling rates for the pre-annealing may be obtained from Figure 7, while Figure 26 plots transient heating data associated with the 600 °C extended testing phase.

#### 3.4.3. Details and Trends

**Figure 27:**
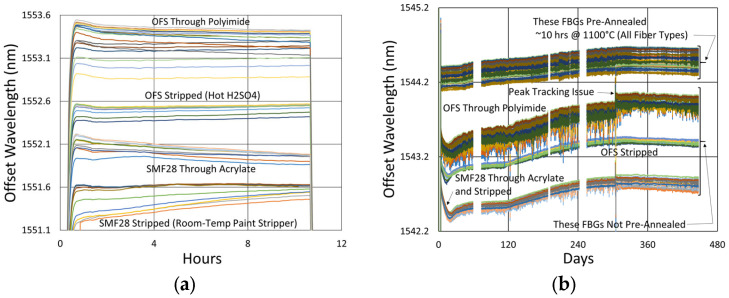
Drift trends of FBGs in SMF28 (SMF) and SMT-A1310H (OFS) fibers subjected to (**a**) 1100 °C pre-annealing and (**b**) 600 °C extended testing.

**Figure 28:**
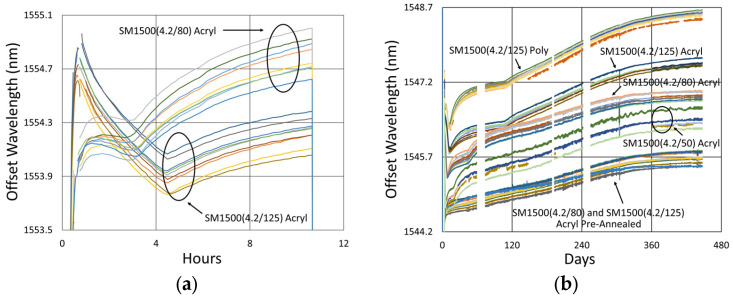
Drift trends of FBGs in SM1500 4.2 µm core fibers through acrylate (Acryl) and polyimide (Poly) coatings subjected to (**a**) 1100 °C pre-annealing and (**b**) 600 °C extended testing.

**Figure 29:**
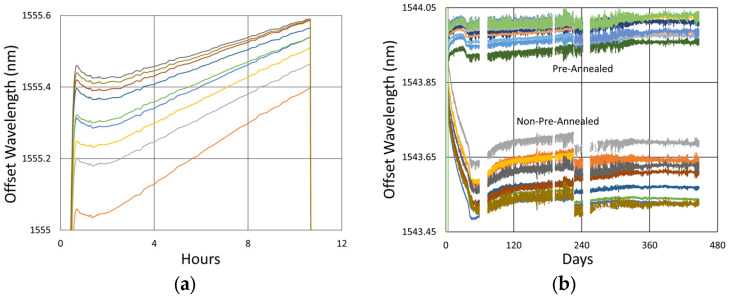
Drift trends of FBGs in stripped OFS A1310 400 µm clad fiber subjected to (**a**) 1100 °C pre-annealing and (**b**) 600 °C extended testing.

**Figure 30:**
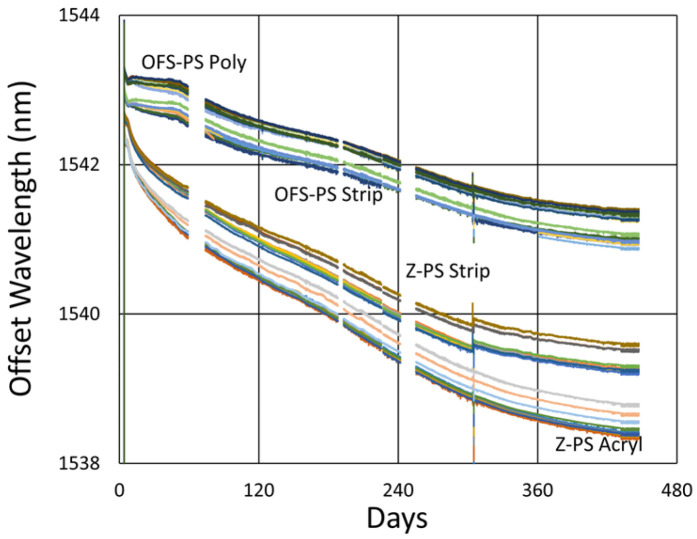
Drift trends of FBGs annealed at 600 °C in pure silica core SMP-E1550H2 (OFS-PS) and Sumitomo Z Fiber (Z-PS) through acrylate (Acryl) and polyimide (Poly) coatings or with the coating stripped (Strip).

In Figure 27, Figure 28, Figure 29 and Figure 30, one can see that for this temperature also, some variation between the devices is present during both pre-annealing and extended testing. In this case, the behaviors of different fiber configurations are somewhat distinct, indicating that the alignment and writing processes are sufficiently uniform and repeatable so as to observe these subtle differences with consistency.

From the long-term results at this temperature, pre-annealing appears to make the drift slopes slightly more gradual and uniform, but in either case, except for the pure silica core fibers, at 600 °C, generally a very long annealing time is necessary (~330 days in SMF28 and SMT-A1310H) before transitioning from red drift to blue drift.

**Figure 31:**
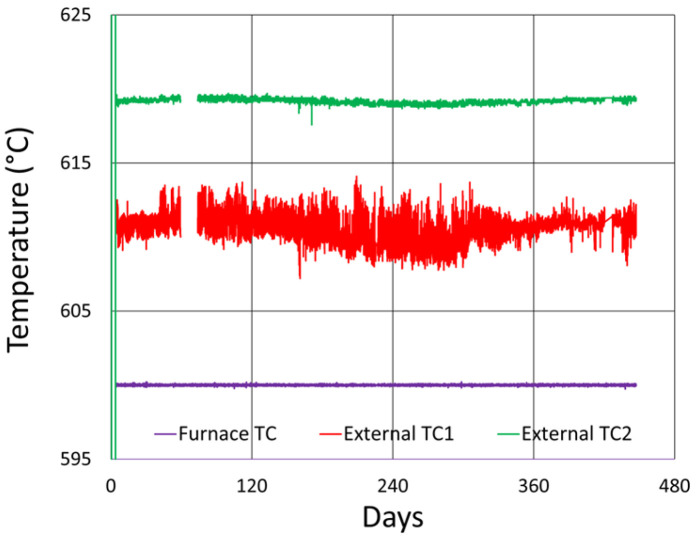
Relative thermocouple drift for 600 °C annealing test.

Figure 31 presents a zoomed view of the thermocouple data. Once again, the error of the furnace thermocouple is expected to be similar to the relative variation measured by the external thermocouples.

The measured drift of both external thermocouples, in both cases, appears to be consistent and small, with a total variation less than 2.2 °C for TC2 and 6.9 °C for TC1. Once again, it is generally much less than that needed to significantly contribute to the observed wavelength drifts, except in the case of the 400 µm clad fiber (Figure 29). Nonetheless, the trends are different, indicating that even for that fiber, there are other mechanisms involved.

## 4. Discussion

**Figure 32:**
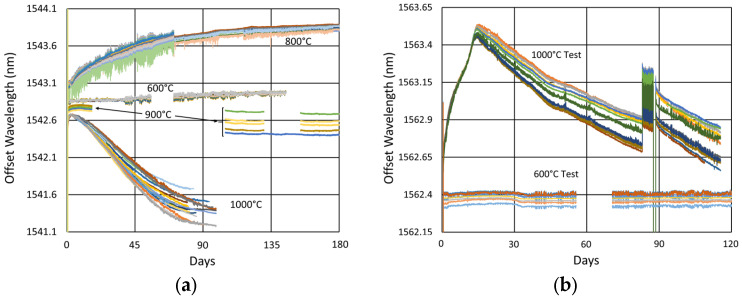
Comparison of long-term wavelength drift at different temperatures for (**a**) SMF28/SMT-A1310H fibers [23] and (**b**) OFS A1310 400 µm clad fiber.

So how do these results fit together? In Figure 32, we first examine the effect that the annealing temperature has on the wavelength drift generated in standard 125 µm and 400 µm clad optical fibers.

As illustrated in Figure 27 and again in Figure 32a, for the 125 µm clad fibers, even at 600 °C, a very slight thermal drift persists, even after a year. At 800 °C, substantial and prolonged red drift is as also present, while at 1000 °C, we see a much more rapid transition from red drift to significant blue drift, together with substantial erasure, occurring over 3 months. At 900 °C, in spite of the missing data, there appears to be a balance between the 800 °C and 1000 °C trends, with a much more stable and moderate rate of blue drift.

Although the 400 µm clad fiber was only tested at two temperatures, in Figure 32b it exhibits similar trends, with an even more gradual and prolonged red drift at 600 °C and significant blue drift at 1000 °C.

The observations confirm that in general for the Ge-doped fibers at each tested temperature, there is a red-drift followed by a blue-drift phase, something that has been reported before [4].

The red drift is associated with increasing mode refractive index and fiber tension, which effectively increase the grating period. It is best explained by some combination of erasure of a negative induced index modulation or by the relief of compressive residual stresses generated and frozen in during the fiber drawing process [4,21]. Devitrification or crystallization of the silica is thought to be a much less likely possibility, as it is generally considered to be very small even at 1100 °C [4,32].

Generally, pre-annealing FBGs prior to extended testing improved their initial response and consistency. This is most evident in Figure 27b, Figure 28b and Figure 29b, where the pre-annealed slopes are more linear over time, with fewer discontinuities. Nonetheless, over the long term, even devices that have not been pre-annealed tend to stabilize, and this occurs more rapidly at higher temperatures. Still, because in practice annealing cannot remove thermal stresses [21], unless they can be eliminated through fiber design, a degree of wavelength drift will always be present and need to be managed.

The more gradual blue drift is associated with a decreasing mode index, which apart from erasure of a positive induced refractive index seems most consistent with dopant diffusion and thermal expansion of the fiber core [4]. This lowers the mode index of the fiber, effectively reducing the grating period and shifting the Bragg resonances to lower wavelengths.

All of these mechanisms accelerate with increasing temperature in line with the faster rollover times present as the test temperature was increased from 600 °C to 1000 °C.

**Figure 33:**
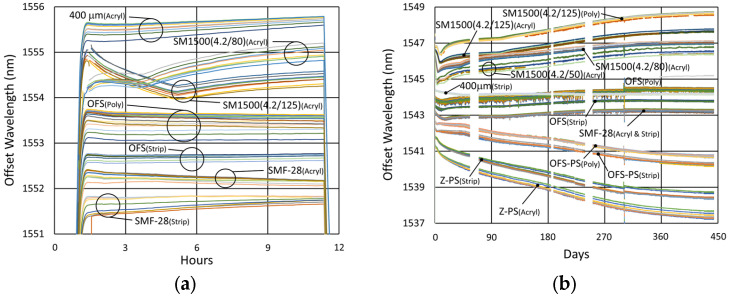
Thermal drift comparison of FBGs without pre-annealing written in SMF-28, SMT-A1310H (OFS), SM1500(4.2/125), SM1500(4.2/80), OFS A1310 400 µm clad (400 μm), SMP-E1550H2 (OFS-PS) and Z Fiber (Z-PS) through acrylate (Acryl) and polyimide (Poly) coatings or with the coating stripped (Strip) at (**a**) 1100 °C [31] and (**b**) 600 °C.

**Figure 34:**
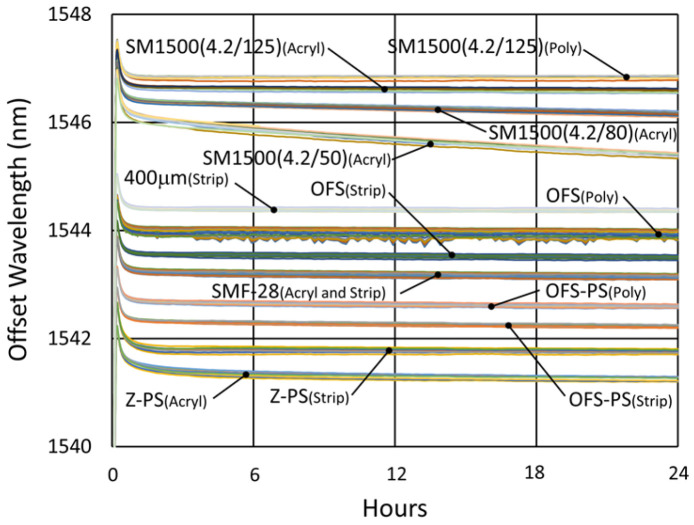
Initial response of FBGs depicted in Figure 33b.

**Figure 35:**
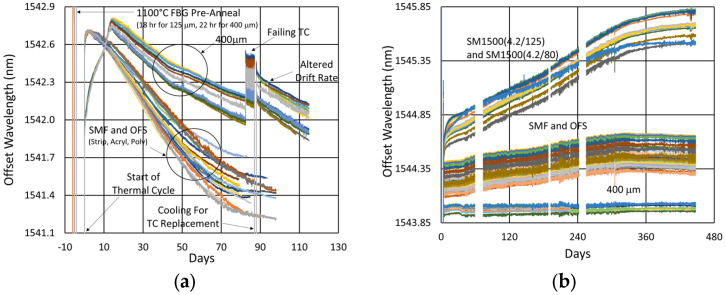
Thermal drift comparison of pre-annealed FBGs in various fibers: (**a**) SMF-28 (SMF) and SMT-A1310H (OFS) contrasted with OFS A1310 400 µm clad (400 μm) fiber subjected to 1000 °C following pre-annealing at 1100 °C for ~20 h [31]; (**b**) SM1500(4.2/125), SM1500(4.2/80), SMF-28 (SMF), SMT-A1310H (OFS) and OFS A1310 400 µm clad (400 μm) fibers subjected to 600 °C following pre-annealing at 1100 °C for ~10 h.

In Figure 33, Figure 34 and Figure 35, we next compare performance differences for the various fibers, observing that they generally produce noticeably distinct wavelength drift trends in response to temperature. This is not surprising since the geometry and dopant concentrations of each waveguide are distinct, leading to different rates and magnitudes of stress relaxation and dopant diffusion, which alter the waveguide effective refractive indices.

In Figure 33b, contrary to the general trend observed for all of the tested Ge-doped fibers, the pure silica core fibers did not exhibit red drift but immediately drifted towards lower wavelengths. This is further confirmed by examining the initial response shown in Figure 34, which appears very similar to the other fibers.

This difference is explained by recalling that the residual stress is made up of thermal, draw-induced and photo-induced components that depend on the fiber structure and materials. For standard fibers with a Ge-doped core and pure silica cladding, residual thermal stresses and draw-induced stresses act in opposition [21]. On the other hand, for the pure silica core fibers tested, the net residual stress is evidently tensile, producing blue drift immediately as these stresses are relieved.

Beyond this, Figure 33b also demonstrates that while the behavior of each fiber is distinct, for similar fibers like SMF28 and SMT-A1310H or in the case of SM1500 fibers having different cladding diameters, over the longer term, the drift rates of similar fibers tend toward a common value.

In Figure 35a, more substantial differences in performance are clearly seen in the 400 µm clad fiber relative to the standard SMF-28 and SMT-A1310H fibers. In particular, one observes that the 400 µm fiber exhibits longer and more gradual red-drift and blue-drift rates. This same tendency is shown in Figure 35b, as these fibers are again compared alongside SM1500(4.2/125) and SM1500(4.2/80).

While the cladding diameter difference between the SM1500 fibers did not produce a significant drift rate difference, something about these fibers increased their drift rates substantially relative to the other fibers tested. One notable distinction is that the SM1500 fibers have significantly higher Ge doping (~20% vs. ~4%), and while it has been observed that the Ge doping in itself “only has a limited impact on thermal stability” of Type II modifications in GeO_2_-SiO_2_ binary glasses [33,34,35], its localization to the fiber core does create a greater degree of material mismatch between the core and cladding of the SM1500 fibers. This will produce larger thermal and draw-induced residual stresses, which will drive movement and relaxation over time, leading to faster drift rates, particularly at higher temperatures.

Finally, using linear regression, in Table 2, the average wavelength drift rates are summarized for the various fibers and annealing temperatures. When a transition has been made from red drift to blue drift, the regression is restricted to the blue-drift portion of the data.

## 5. Conclusions

In conclusion, it is apparent from the test results that the dynamics of the red drift and blue drift are complex, temperature-dependent and different from one fiber type to another, with pure silica core fibers bypassing the red-drift phase altogether.

Pre-annealing FBGs are shown to improve their initial response and consistency, likely through the relief of residual stress in the fiber. But in practice, a degree of wavelength drift will always be present, since thermal expansion mismatch between the core and cladding will produce new thermal stresses whenever temperature change occurs.

In SMF-28, 900 °C offers a sweet spot for stability. Although there are structural differences in these gratings (due to greater nanopore disruption), their comparable performance in Figure 8b, combined with a reasonable expectation that the 900 °C drift should lie between the red drift at 800 °C and the blue drift at 1000 °C, gives confidence that this sweet spot really exists.

Generally, larger drifts were observed for the SM1500 fibers owing to their higher Ge-doping as well as the larger thermal expansion mismatch of their core and cladding. For these fibers, variations in cladding diameter did not significantly alter their isothermal drift response.

On the other hand, much smaller drifts were observed for the 400 μm clad fiber. We speculate this could be due to the impact of its much larger cross-sectional area on the lowering of tensile stress while also slowing the cooling of the core during fiber drawing. This would reduce draw-induced residual stresses.

Finally, it is emphasized that beyond grating lifetime considerations, it is important to be mindful of the fact that subject to required levels of accuracy, wavelength drift specific to the chosen waveguide and desired test temperature will limit the maximum test time available before sensor re-zeroing or recalibration is required. These results offer a uniquely robust and long-term set of measurements, providing new guidance for the design of high-temperature sensing systems, and will greatly help in optimizing fiber selection as well as estimating recalibration intervals necessary for ensuring system accuracy.

## Figures and Tables

**Table 2:** Average wavelength drift rates of FBGs in various fibers (pm/day).

Temperature °C	SM1500	SMF/OFS ^1^	400 μm ^2^	OFS-PS ^3^	Z-PS ^4^
1000 ^5^	~	−16	−7	~	~
1000	~	−12 [4]	~	~	~
900 ^6^	~	−0.77	~	~	~
800 ^5^	~	1.5	~	~	~
800	~	2.1	~	~	~
600 ^5^	2.7	0.84	0.027	~	~
600	5.0	1.6	−0.29	−5.1	−10

^1^ SMF-28 (SMF), SMT-A1310H (OFS); ^2^ OFS A1310 400 μm clad (400 μm); ^3^ SMP-E1550H2 (OFS-PS); ^4^ Z Fiber (Z-PS); ^5^ subjected to 1100 °C pre-annealing; ^6^ subjected to 1000 °C pre-annealing.

## Data Availability

The original contributions presented in this study are included in the article.
